# A Visit to the Front

**Published:** 1920-05-08

**Authors:** 


### A Visit to the Front.

### By the Official Conductor.

All sorts and conditions of suffering humanity, provided only that there was need for help, appealed strongly to the sympathetic nature of Sir Henry Burdett. In 1917, when the public was deeply concerned and full of anxiety for the wounded on our Western Front, with the co-operation of Sir Alfred Keogh, the Director-General of the Army Medical Service, Sir Henry undertook, under the guidance of Col. S. Westcott, C.B., C.M.G., the D.D.M.S. of the Army Corps, a pilgrimage to the battle area, in order personally to study the conditions of evacuation and treatment of our wounded soldiers.

#### From Battlefield to Hospital Bed.

This mission he carried through with characteristic thoroughness and bravery. Starting at the actual firing-line, he traced the course of the wounded through the trenches and the advanced and main dressing-stations to the clearing-stations.

It was during this journey that Sir Henry himself narrowly escaped becoming a casualty. When passing through a short open space the party was observed by the enemy and shelled, one of five shells passing so near his head as to displace his helmet.

The rapid strides which had been made in the comfort and speed of evacuation, and in the arrangements for early surgical treatment at the clearing-stations, impressed Sir Henry more than anything. These clearing-stations, at the time of his visit, had already been pushed up to the limit of enemy gunfire, and had been equipped in the manner of the best London hospitals. Of these he visited a large number, with an aggregate of many thousands of beds. The articles already published in The Hospital arose out of his visit. It was hoped that they would exercise a soothing influence upon the public mind at the time of their appearance. The first article was published in The Hospital of February 9, 1918, when, as will be remembered, there was considerable public anxiety.

